# Enhancing Osteoblast Differentiation from Adipose-Derived Stem Cells Using Hydrogels and Photobiomodulation: Overcoming In Vitro Limitations for Osteoporosis Treatment

**DOI:** 10.3390/cimb46070379

**Published:** 2024-06-25

**Authors:** Daniella Da Silva, Anine Crous, Heidi Abrahamse

**Affiliations:** Laser Research Centre, Faculty of Health Sciences, University of Johannesburg, P.O. Box 17011, Johannesburg 2028, South Africa; 216040611@student.uj.ac.za (D.D.S.); acrous@uj.ac.za (A.C.)

**Keywords:** osteoporosis treatment, stem cell regenerative therapy, adipose-derived stem cells, osteogenic induction, three-dimensional cell culture, photobiomodulation, in vitro–in vivo relationship

## Abstract

Osteoporosis represents a widespread and debilitating chronic bone condition that is increasingly prevalent globally. Its hallmark features include reduced bone density and heightened fragility, which significantly elevate the risk of fractures due to the decreased presence of mature osteoblasts. The limitations of current pharmaceutical therapies, often accompanied by severe side effects, have spurred researchers to seek alternative strategies. Adipose-derived stem cells (ADSCs) hold considerable promise for tissue repair, albeit they encounter obstacles such as replicative senescence in laboratory conditions. In comparison, employing ADSCs within three-dimensional (3D) environments provides an innovative solution, replicating the natural extracellular matrix environment while offering a controlled and cost-effective in vitro platform. Moreover, the utilization of photobiomodulation (PBM) has emerged as a method to enhance ADSC differentiation and proliferation potential by instigating cellular stimulation and facilitating beneficial performance modifications. This literature review critically examines the shortcomings of current osteoporosis treatments and investigates the potential synergies between 3D cell culture and PBM in augmenting ADSC differentiation towards osteogenic lineages. The primary objective of this study is to assess the efficacy of combined 3D environments and PBM in enhancing ADSC performance for osteoporosis management. This research is notably distinguished by its thorough scrutiny of the existing literature, synthesis of recent advancements, identification of future research trajectories, and utilization of databases such as PubMed, Scopus, Web of Science, and Google Scholar for this literature review. Furthermore, the exploration of biomechanical and biophysical stimuli holds promise for refining treatment strategies. The future outlook suggests that integrating PBM with ADSCs housed within 3D environments holds considerable potential for advancing bone regeneration efforts. Importantly, this review aspires to catalyse further advancements in combined therapeutic strategies for osteoporosis regeneration.

## 1. Introduction

Osteoporosis and similar degenerative bone conditions present substantial obstacles to healthcare systems worldwide, given their widespread occurrence and detrimental effects on quality of life [1]. Present therapeutic approaches frequently focus on symptom alleviation and do not offer holistic remedies for the restoration of compromised or deteriorated bone tissue [2]. This encourages the investigation of pioneering methods, such as regenerative medicine using stem cells (SCs), to transform the treatment paradigm [3], offering fresh opportunities for addressing a range of diseases and injuries [4]. Stem cells are central to these advancements, holding the potential to specialise into distinct cell types and aid in the repair and regeneration of tissues [5]. Among the various origins of SCs, adipose-derived stem cells (ADSCs) have attracted significant interest due to their accessibility, plentiful supply, and remarkable ability to differentiate [6]. While ADSCs possess significant potential, their inherent tendency toward adipogenic differentiation necessitates the strategic application of differentiation inducers (DIs) to redirect them toward osteogenic lineages [7]. Furthermore, three-dimensional (3D) cell culture plays a crucial role in promoting the enhanced maturation of ADSCs into osteoblasts by providing a more physiologically relevant microenvironment [8]. Additionally, photobiomodulation (PBM) is known to improve cell proliferation and differentiation [9]. Nevertheless, there are various methodologies for this technique that have not yet been established and must be further explored and developed to make a noticeable difference in stem cell therapy [10]. This review investigates the diverse DIs utilised to guide the fate of ADSCs, emphasizing the critical role of precise control over lineage-specific differentiation for achieving effective regenerative results, while also delving into potential transformation by employing 3D cell culture methods to encourage ADSCs to adopt an osteoblastic destiny, emphasizing their application in tackling osteo-degenerative conditions. Moreover, PBM enhances the differentiation and proliferation potential of ADSCs. Finally, we explore the osteogenic differentiation potential of ADSCs under the combined influence of osteogenic DIs, 3D cell culture, and PBM as a potential therapeutic strategy for addressing osteo-degenerative diseases. This article contributes to the advancing field of regenerative medicine and offers promising prospects for future therapeutic interventions in osteo-degenerative diseases by investigating the nuances of differentiation induction, harnessing the benefits of ADSCs, and acknowledging the importance of biomechanical and biophysical stimulation, with the literature review relying on databases such as PubMed, Scopus, Web of Science, and Google Scholar.

## 2. Osteo-Degenerative Disease and Current Therapeutic Strategies

**Figure 1:**
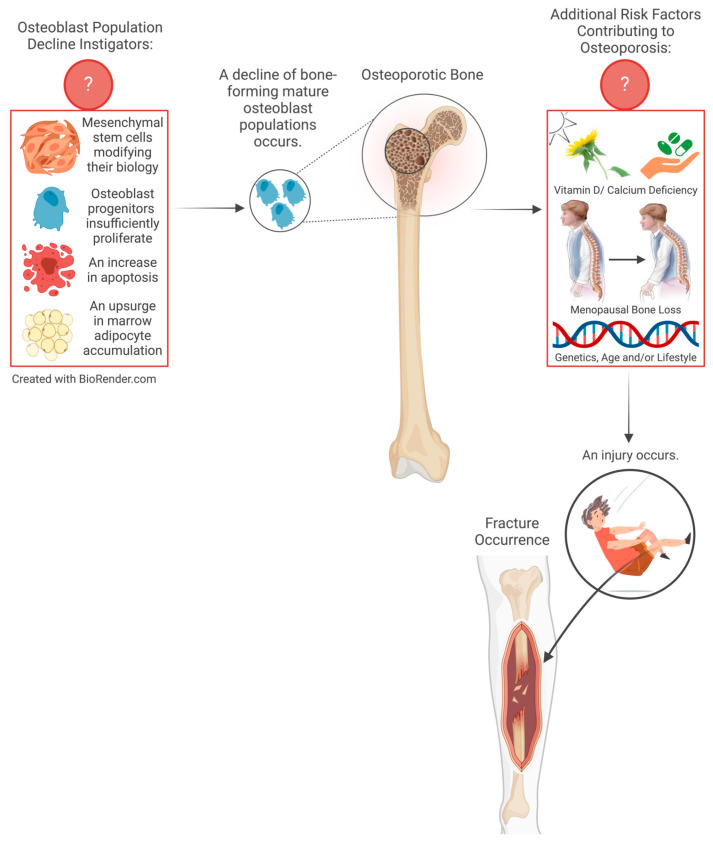
The underlying instigators that trigger the osteoporotic process are often accompanied by probable risk factors that exacerbate the progression of osteoporosis, leading to an increased likelihood of fatal fractures.

Osteoporosis is a degenerative disease characterised by the loss of bone mass and deterioration of bone tissue, leading to an increase in bone fragility and an increased risk of fractures [11]. Osteoporosis occurs because of a decrease in resident bone marrow mesenchymal stem cells (MSCs) as well as decreased MSC function, resulting in MSCs’ failure to appropriately proliferate, differentiate, and, as a result, form bone [12]. This disease primarily affects females and older individuals, particularly postmenopausal women, although it can also develop in men and younger individuals due to various factors such as endocrine imbalances, genetic factors, nutrition, and inactive lifestyles [13]. Figure 1 illustrates both the recognised instigators and risk factors associated with osteoporosis. There are multiple diagnostic modalities for osteoporosis, including bone density scans like dual-energy X-ray absorptiometry (DXA), which assess bone mineral density [14]. Another non-invasive method is quantitative ultrasound (QUS), especially useful for evaluating bone health in peripheral areas like the heel [15]. Bone profile blood tests are also valuable for measuring markers of bone turnover, providing insights into bone metabolism [16]. Additionally, advanced imaging techniques such as magnetic resonance imaging (MRI) and computed tomography (CT) scans offer detailed assessments of bone structure and integrity [17]. The prevalence of osteoporosis varies by country, but globally, it is estimated that over 200 million individuals suffer from osteoporosis [18]. Reciprocally, osteoporotic fractures are a growing health concern, with an estimated 8.9 million fractures occurring annually worldwide [19].

Osteoporosis and other degenerative bone disease treatment strategies regularly employ bisphosphonates, hormone replacement therapy (HRT), selective oestrogen receptor modulators (SERMs), parathyroid hormone (PTH) analogues, or receptor activator of nuclear factor-kappa B ligand (RANKL) inhibitors (Table 1). Treatment strategies for osteoporosis tend to be individualised, depending on numerous influences, such as the patient’s age, sex, bone density, fracture risk, and overall health [20]. Bisphosphonates are the first line of antiresorptive medications utilised for osteoporosis treatment, which work by inhibiting the activity of bone-resorbing cells called osteoclasts, thereby reducing the loss of bone at the same time as preserving the bone density [21]. Unfortunately, bisphosphonates medications produce agonizing ramifications of gastrointestinal discomfort, atypical fractures, muscle and joint pains, hypocalcaemia and, in extremely rare instances, osteonecrosis of the jaw may occur [21]. Hormone replacement therapy, specifically oestrogen replacement therapy, is used to treat osteoporosis in postmenopausal women by decelerating bone loss along with diminishing fracture incidence risk [22]. A patient is prescribed HRT based on their specific circumstances and medical history as complications of blood clots, generation of breast cancer, fatal strokes, breast tenderness, and swelling and dementia are associated with HRT [21]. Selective oestrogen receptor modulators, like raloxifene and tamoxifen, are a treatment strategy predominantly used in postmenopausal osteoporosis [11]. This treatment selectively targets oestrogen receptors in distinct tissues, preventing bone loss and fracture occurrence [22]. Selective oestrogen receptor modulators are an alternative treatment to HRT as the risk of invasive breast cancer is diminished [13]. Despite this, leg cramps, venous thromboembolism, endometrial alterations, musculoskeletal discomfort, gastrointestinal distress, and an increased risk of strokes remain as adverse effects associated with SERM therapy [23]. Parathyroid hormone synthetic analogues, namely teriparatide and abaloparatide, are administered as daily injections to assist in the stimulation of bone formation, resulting in enhanced bone density and decreased fracture risk [24]. The adverse effects of PTH treatment restricting its use as a therapeutic method include hypercalcaemia, injection site responses, leg spasms, and orthostatic hypotension [13,21]. Additionally, PTH treatment might elevate the risk of osteosarcoma in individuals with Paget’s disease of bone, unexplained alkaline phosphatase elevations, paediatric and young adult patients with open epiphyses, or those with a history of external beam or implant radiation therapy affecting the skeleton [25]. Receptor activator of nuclear factor-kappa B ligand inhibitors, for example denosumab, are novel medications which block the receptor activator of nuclear factor-κB protein, liable for bone breakdown [26]. Despite RANKL inhibitors being effective in lessening fracture occurrence in addition to augmenting bone density, these medications have possible side effects of injection site-related infections, hypocalcaemia, musculoskeletal pains, osteonecrosis of the jaw, and atypical fractures, limiting preference for them as a treatment strategy [27]. Non-adherence to anti-osteoporosis medication, influenced by patient-specific barriers like forgetfulness, concerns about side effects, and perceived treatment effectiveness, as well as system-related obstacles such as medication costs and inadequate patient education, poses as an additional significant challenge to the effectiveness of current treatment strategies for osteoporosis [28]. Moreover, the limited efficiency and adverse side effects of existing antiresorptive and anabolic drugs have heightened the need for the development of novel therapeutic techniques to enhance bone regeneration in osteoporosis patients.

## 3. Regenerative Medicine and Tissue Engineering Advancements

The repairing, replacement, or regeneration of damaged and/or diseased tissues and organs is the therapeutic aspiration of regenerative medicine strategies [34]. Tissue engineering, which predominantly combines stem cell-based treatment, 3D methods, and differentiation inducers, is a considerable component of regenerative medicine, as well as additional techniques involving gene therapy and immunomodulation [35]. Regenerative stem cell-based therapies currently face numerous challenges, including cell sourcing and isolation, control over cell differentiation fate, integration of transplanted cells, potential immune rejection in allogeneic cell therapies, and ethical conflicts [36]. Nonetheless, because regenerative medicine is a rapidly evolving field, research is ongoing to address the limitations of regenerative stem cell-based techniques and improve the efficacy of this regenerative therapeutic strategy. Tissue engineering is a multi-disciplinary section of regenerative medicine that integrates stem cells, bioactive molecules, and biomaterial scaffolds in the laboratory to develop functioning tissues and/or organs for therapeutic transplantation [35]. Stem cells are an essential component of tissue engineering since they assist in the development and function of the engineered tissue [34]. Stem cells can be derived from a variety of sources, including bone marrow, amniotic cells, adipose tissue, the umbilical cord, and placental tissue [4]. Stem cells are undifferentiated cells that possess the ability to differentiate into specialised cell types and self-renew, being an asset for tissue engineering-based treatments [34]. Stem cell regenerative therapy utilises two main types of SCs: pluripotent stem cells (PSCs), such as embryonic stem cells (ESCs) and induced pluripotent stem cells (iPSCs), and adult stem cells (ASCs), including MSCs [4], whereby each cell type possesses distinct characteristics and potential therapeutic uses, along with limitations [37], as indicated in Table 2. Derived from the innermost cell mass of an early stage developing embryo, specifically from a blastocyst, ESCs exhibit remarkable differentiation potential [38]. Desired for diverse therapeutic applications, their capacity to generate cells from all three germ layers, produce an abundance of cells, and serve as an endless source of stem cells enhances their overall appeal [39]. Conversely, ESCs raise significant ethical conflict due to the utilisation of human embryos as well as the significant probability of tumorigenesis if cells have not undergone complete differentiation [38]. Adult somatic cells are reprogrammed to a pluripotent state comparable to ESCs to form iPSCs, which have similar properties to ESCs, such as their capacity to develop into numerous cell types, often overcoming the ethical disputes associated with the use of embryonic tissues [39]. Unfavourably, the iPSC reprogramming procedure is time-consuming, technically difficult, and possesses the potential to cause genetic and epigenetic defects liable for tumorigenesis [40]. Adult stem cells are undifferentiated cells that are present in many bodily tissues and are essential for tissue repair and homeostasis [41]. They can either be unipotent, meaning they can only produce one type of cell, or multipotent, meaning they can develop into specific cell types within the tissue from where they originated [37]. Adult stem cells can be easily harvested from the patient’s own tissue and do not provide the ethical challenges that are often associated with SCs, particularly ESCs and iPSCs; however, ASCs have a few drawbacks, such as a limited ability to differentiate compared to iPSCs, challenges obtaining enough ASCs based on isolation site, and a reduction in SC function [42]. Mesenchymal stem cells are adult multipotent stem cells that can differentiate into ectodermal lineages like neurocytes and endodermal lineages like hepatocytes, as well as mesodermal lineages including osteocytes, adipocytes, and chondrocytes [43]. The bone marrow, adipose tissue, amniotic fluid, endometrium, dental tissues, umbilical cord, and Wharton’s jelly are among the tissues from which MSCs can be harvested [44]. Mesenchymal stem cells are a useful tool in the treatment of chronic diseases because of their multilineage potential, immunomodulation, and release of anti-inflammatory molecules [45]. Research is ongoing to optimise the use of stem cell-based regenerative treatments and address their limitations, with the goal of increasing their safety and effectiveness. Additionally, DIs are used to promote cell differentiation, proliferation, and tissue development as they play a vital role in influencing cellular activity [45]. The 3D porosity structure of scaffold materials encourages cell survival, adhesion, and interaction [46]. Scaffolds, designed to emulate the extracellular matrix (ECM) deposition in natural tissues and facilitate cell organization and development, utilise a range of materials including natural ones like collagen, gelatine, and alginate, as well as synthetic polymers such as poly(lactic-co-glycolic acid) (PLGA), polyethylene glycol (PEG), and decellularized tissue scaffolds [47]. Tissue engineering materials should be biocompatible, meaning they should not be toxic to cells or trigger an immune response, and biodegradable since the scaffold should gradually break down as new tissue develops [48]. The development and functionality of tissues can be enhanced by simulating the mechanical forces encountered by natural tissues using apparatus like bioreactors and mechanical stimulation techniques like fluid flow or mechanical stretching [49]. The successful development and application of tissue engineering techniques hinge on essential elements, encompassing engineered tissue integration, vascularization, and overcoming challenges such as replicating the intricate structures and functions of natural tissues, addressing limitations in the vascularization of engineered tissues, ensuring engineered tissue stability and durability, and navigating regulatory challenges for clinical approval, collectively establishing tissue engineering as a novel therapeutic strategy [50]. Notwithstanding these present difficulties, tissue engineering presents the possibility of creating customised scaffolds for intricate tissue structures like blood vessels, cartilage, skin, bone, liver, heart, kidney, and/or nerves. Additionally, it offers the possibility of producing tissue constructs on a large scale and reducing the risk of immunological rejection in autologous tissue engineering [40].

## 4. Adipose-Derived Stem Cells as the Favourable Cell Source

**Figure 2:**
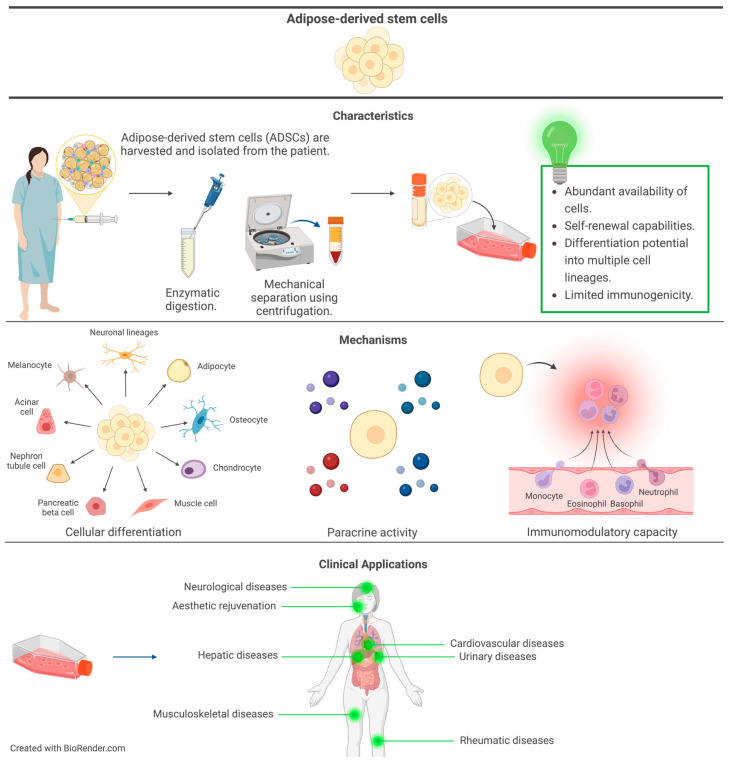
Adipose-derived stem cells are easily harvested and isolated using established methods. They possess favourable traits such as abundant cell supply, self-renewal capabilities, and the ability to differentiate into multiple cell types, while also exhibiting limited immunogenicity. The mechanisms of these cells enable differentiation into various cell types and provide immunomodulatory support, contributing to paracrine activity. Currently, due to their advantageous characteristics, adipose-derived stem cells are proving valuable in a wide range of regenerative therapeutic applications.

Stem cells derived from adipose tissue through minimally invasive methods such as liposuction or fat tissue excision, known as ADSCs, represent a subtype of MSCs that possess the ability to undergo differentiation into various cell lineages [54]. Due to their ease of isolation, diverse therapeutic potential, and abundance when compared to other SC sources like bone marrow, ADSCs have garnered significant interest in regenerative medicine [55]. Adipose-derived stem cells, distinguished by their notable self-renewal capacity and the ability to thrive in culture while retaining their SC characteristics, present an abundant and sustainable cell source for regenerative purposes [7]. Adipocytes, osteocytes, chondrocytes, myocytes, and neuronal cells are among the diverse cell lineages that ADSCs can differentiate into, showcasing their high differentiation capacity and rendering them valuable for the repair of various tissues and organs [56]. Harvested from the patient’s own adipose tissue and endowed with immunomodulatory capabilities, ADSCs, through the production of factors that reduce inflammation and regulate the immune system, exhibit potential benefits in treating autoimmune illnesses and alleviating tissue damage caused by immunological responses, while simultaneously minimizing the risk of immunological rejection [57]. Figure 2 presents the depiction of preparation, characteristics, mechanisms, and applications in tissue regeneration. The arrangement and properties of ADSCs exhibit variability among individuals and within adipose tissue depots, potentially influencing the quality and consistency of ADSCs obtained from different sources [54]. Though ADSCs can differentiate into various cell types, their potential for differentiation may be more limited compared to ESCs or iPSCs, with variations observed based on the methods and settings employed for differentiation [7]. Ensuring consistency and safety in clinical applications necessitates standardised protocols and quality control measures in the isolation, growth, and characterisation of ADSCs; although ADSCs have demonstrated potential in preclinical and early clinical research, long-term safety and effectiveness data are still in the initial stages of investigation, warranting additional research and clinical studies to ascertain the optimal methods, dose, and long-term effects of ADSC-based therapies [58]. Furthermore, an obstacle in effectively utilizing ADSCs is their innate tendency toward adipogenic differentiation, which can impede endeavours to steer them towards desired lineages like osteogenic or chondrogenic [59]. To overcome this challenge and fully harness the potential of ADSCs for tissue regeneration, it is imperative to strategically utilise DIs. Differentiation inducers function as biochemical signals redirecting ADSCs from their default adipogenic fate towards alternative lineages, promoting the expression of specific transcription factors and signalling pathways to effectively reprogram them towards the desired cell fate [60].

## 5. Utilizing Differentiation Inducers to Direct Cell Fate

Cell fate direction is the process of steering undifferentiated SCs towards lineages or cell types by using DIs, which enable SCs to differentiate into cell types relevant to the intended therapeutic purpose [61]. Differentiation inducers offer a standardised and consistent method for guiding SC differentiation by consistently inducing specific molecular and cellular alterations, mimicking signals present during natural developmental processes, thereby increasing the effectiveness of SC differentiation by yielding larger populations of the desired cell type and reducing the differentiation time [62]. The range of possible applications can be increased by controlling the type and concentration of DIs, which enables the regulation of SC lineage commitment and the creation of many cell types from a single population of SCs [61]. Recreating the intricate in vivo differentiation processes, which involve a combination of signalling molecules, DIs, and physical prompts, using DIs in vitro can be challenging and may not fully replicate the complexity of natural developmental processes [62]. The use of DIs may not consistently lead to thorough and mature differentiation of the target cell type from SCs, as the produced cells might exhibit immature or partially differentiated features, thereby restricting their functionality and application [63]. The quality and uniformity of the generated cell populations may vary depending on the efficiency and efficacy of differentiation [63]. The incomplete understanding of the processes governing SC differentiation and the conditions required for successful differentiation into specific cell types for all cell types limits the ability to optimise and modify differentiation protocols, necessitating further investigation to achieve desired results [64]. The careful selection and optimisation of DIs is crucial because these compounds can impact diverse cellular processes and may induce off-target effects, leading to the formation of unexpected cell types or undesirable cellular behaviour; thus, minimising such consequences is essential for effective SC differentiation [65]. Although DIs have played a crucial role in directing ADSCs towards preferred lineages like osteogenic or chondrogenic, there is an increasing acknowledgment of the necessity for supplementary approaches to enhance differentiation regulation. Three-dimensional scaffolds and hydrogels, which serve as structural cellular environments, are increasingly recognised as effective tools for guiding ADSC fate and counteracting their inherent tendency toward adipogenesis [47]. These scaffolds not only offer a physical framework for cell attachment and proliferation but also replicate the intricate nature of the native tissue microenvironment, providing spatial cues that influence cellular behaviour and commitment to specific lineages [66]. The integration of structural cellular environments alongside traditional DIs has enormous potential to fully unleash the regenerative capabilities of ADSCs, providing novel avenues for effectively addressing tissue defects and degenerative diseases with heightened precision and efficacy.

## 6. Three-Dimensional Cell Culture as a Biomechanical Stimulant for Advanced Cellular Development

**Figure 3:**
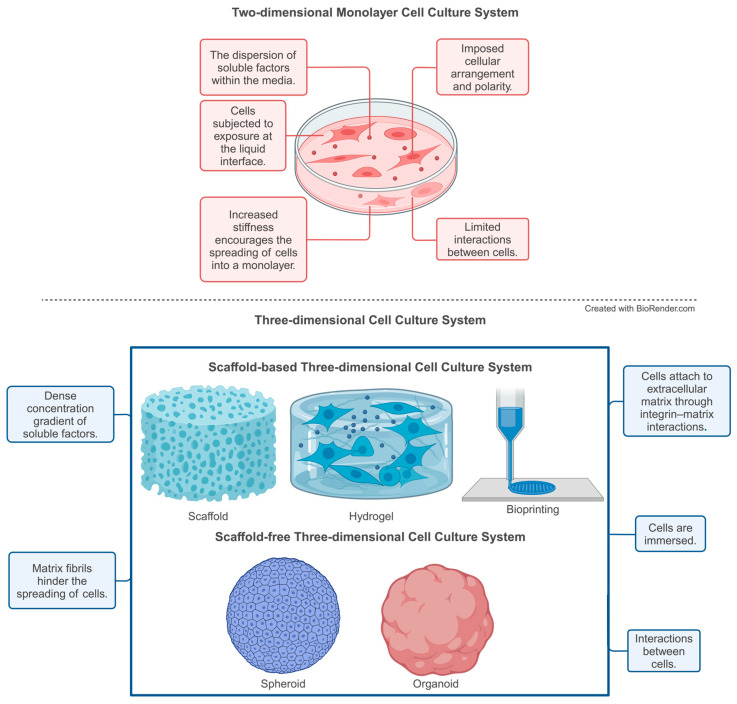
Comparison between two-dimensional and three-dimensional cell culture systems.

Cells can thrive and engage in a 3D environment rather than on the frequently employed 2D surfaces, with the goal of 3D cell culture techniques being to replicate the intricate microenvironment found in tissues and organs [67]. The scaffold functions as a 3D framework facilitating cell adhesion, proliferation, and tissue development, enabling the creation of tissue-engineered structures for organ regeneration in a supportive and biomimetic environment [68]. Additionally, 3D cell culture techniques assist in the understanding of cell-to-matrix and cell-to-cell interactions and evaluate medication efficacy and toxicity within a more physiologically realistic setting [69] (Figure 3).

Prominent 3D cell culture techniques incorporate scaffold-based systems, hydrogel-based approaches, spheroids, organoids, and bioprinting (Table 3). The three frequent types of scaffolds are natural scaffolds, synthetic scaffolds, and composite scaffolds, each possessing distinct advantages and drawbacks. Natural scaffolds are derived from biological constituents like collagen, fibrin, alginate, chitosan, or a decellularized ECM [70]. Due to their composition and architecture closely resembling the natural tissue ECM, natural scaffolds offer bioactive components that can enhance cell attachment, proliferation, and differentiation, mimic the biochemical and mechanical characteristics of original tissues, improve cell-to-matrix interactions, and facilitate the formation of complex tissue architectures [71]. Conversely, natural scaffolds face limitations in controlling scaffold characteristics and degradation kinetics, along with challenges related to standardisation and variability in composition and mechanical properties across different batches [72]. Polymers like PLGA, PEG, polycaprolactone (PCL), and poly(vinyl alcohol) (PVA) are frequently employed in the fabrication of synthetic scaffolds, offering customisable properties for diverse shapes and sizes, precise control over scaffold composition, mechanical characteristics, and degradation kinetics, allowing tailored scaffold designs for specific tissue types and applications, and ensuring high reproducibility in production [48]. Nevertheless, synthetic scaffolds face constraints in terms of cell adhesion and bioactivity due to the absence of natural ECM components [73]. Composite scaffolds combine various materials, including natural and synthetic polymers or ceramics, to synergistically leverage their distinct characteristics, creating a combination that mimics both the biochemical and mechanical aspects of the original tissue ECM and enhancing cell adhesion, proliferation, and differentiation [74]. Composite scaffolds present challenges in accurately adjusting the ratios and interactions among different materials, along with concerns related to batch-to-batch variability and standardisation [75]. The current limitations of scaffold materials may include restricted biocompatibility, elicitation of immunological responses, influence on cellular behaviour, alteration of cell phenotype, limited diffusion capabilities, impact on the delivery of nutrients and oxygen to cells, and challenges associated with disintegration kinetics [76]. Hydrogels, which are gel-like scaffolds utilised in 3D cell culture techniques, consist of hydrophilic polymer networks like agarose, alginate, gelatine, or hyaluronic acid, providing a hydrated and biocompatible environment for cells due to their high-water content [47]. Hydrogel-based 3D cell culture environments stand out for their distinct advantages, thanks to their adjustable properties, biomimetic characteristics, and versatility [77]. In contrast to scaffold-based systems, which might offer limited adjustability, hydrogels provide researchers with precise control over parameters like stiffness, porosity, and degradation rate, allowing them to customise the environment to closely mimic specific tissue traits [47]. The inherent biocompatibility and ECM-like hydrated matrix of hydrogels promote cell adhesion, proliferation, and differentiation, thereby maintaining cell viability and functionality and accurately representing cellular responses in physiological conditions [77]. Moreover, hydrogel-based 3D cultures have the capability to accommodate a wide range of cell types, spanning from stem cells and primary cells to tissue-derived cells, facilitating the construction of intricate multicellular structures like spheroids and organoids [78]. This versatility allows researchers to more accurately replicate tissue architecture and cellular interactions, resulting in experimental models that are more physiologically relevant [79]. Additionally, hydrogels can readily integrate bioactive molecules, DIs, and drugs, allowing for controlled release and spatiotemporal regulation of signalling cues to modulate cellular behaviour [80]. Despite the current limitations hydrogels encounter in mechanical strength, stability, nutrient and oxygen diffusion, and maintaining precise control over their characteristics and degradation rates, further investigation is warranted for the optimisation of this beneficial 3D cell culture technique [81]. The cultivation of cells in suspension or embedding them in a gel matrix to form 3D cellular aggregates, known as spheroids and organoids, respectively, involves the creation of clusters of cells known as spheroids or more complex formations comprising various cell types that mimic specific organs termed as organoids [82]. These techniques are employed for simulating tissue formation, disease modelling, medication screening, and personalised medicine [83]. As cells self-organize into more physiologically realistic structures, they enable the study of cellular interactions, tissue morphogenesis, and disease processes in a 3D framework [84]. However, the spheroid and/or organoid 3D cell culture technique has restricted control over size, shape, and cellular composition, reproducibility and variability issues, diffusion difficulties within the structures affecting nutrient and oxygen supply, and long-term culture and maintenance of complex organoids, which can be technically challenging [83]. Bioprinting enables the precise layer-by-layer deposition of cells, biomaterials, and differentiation inducers, allowing for the creation of intricate 3D structures with controlled cell distribution and spatial organization in the formation of sophisticated tissue structures [85]. Bioprinting plays a crucial role in advancing tissue engineering, organ transplantation, drug discovery, and regenerative medicine, as its 3D methodology provides precise control over cell distribution, design, and composition of structures, facilitating the incorporation of diverse cell types and biomaterials to form intricate tissues and organs with circulatory networks [86]. Nevertheless, choosing suitable bio-inks and biomaterials poses challenges, representing a costly and technically demanding process constrained by limited equipment and experience [86]. The choice of a specific 3D cell culture method, with its unique advantages and limitations, is guided by the specific research or application goals, and each of these techniques necessitates additional optimization to ensure reproducibility in successful cell proliferation, differentiation, and physiologically realistic cell-to-cell interaction for therapeutic purposes. Moreover, incorporating mechanical stimulation within these structural platforms enhances differentiation outcomes by mimicking the dynamic mechanical cues found in living tissues [87].

## 7. Further Biomechanical Stimuli to Enhance Cellular Differentiation

The use of physical forces or mechanical signals to accelerate cell growth and direct SCs towards certain cell lineages is referred to as enhanced cellular proliferation and cellular differentiation utilising mechanical stimulants [91]. Mechanical stimulation consists of diverse methods, including fluid flow, stretching, compression, and substrate stiffness [92]. Mechanical stimulants are designed to imitate the mechanical stresses and signals that cells receive in their natural microenvironment [91]. Mechanical stimulation encourages cell proliferation by initiating intracellular signalling pathways involved in cell cycle progression and DNA synthesis [93]. Mechanical forces stimulate cell stretching or straining, which results in the activation of growth hormones and cell cycle-related proteins, boosting cell division and cell proliferation [94]. Mechanical stimuli encourage cytoskeletal re-organization, activate mechano-sensitive signalling pathways, and control gene expression associated with cell lineage commitment, thus furthering directed cellular differentiation [95]. Mechanical stimuli play a crucial role in directing SCs towards specific cell lineages by influencing cell differentiation pathways, while also aiding differentiated cells and tissues in their maturation and functionality [95]. Due to mechanical stimuli, such as cyclic stretching or compression, the cells can align, organise, and develop in a way comparable to natural tissues, resulting in advanced tissue functionality [96]. The intricacies and dynamics of the mechanical characteristics within the extracellular matrix and the microenvironment are notably complex, making the recreation of these dynamic conditions in vitro challenging and potentially inadequate in fully capturing the intricate interactions and signals inherent in natural tissues [96]. The identification of ideal mechanical parameters for various cell types and tissues, such as magnitude, frequency, duration, and method of stimulation, remains a challenge [97]. Mechanical stimulation effectivity is very context-dependent, and optimising these parameters for specific applications necessitates thorough investigation and knowledge of cellular responses. The interaction of mechanical stimuli with biochemical substances and signalling pathways crucial for cell proliferation and differentiation is common, yet the challenge lies in discerning the distinct effects of mechanical stimulation from those of biochemical signals, thereby complicating the assessment of the isolated contributions of mechanical forces [98]. The responses of cells to mechanical stimulation exhibit variability based on their sources and/or individuals, and the efficiency of mechanical stimulation in promoting cellular proliferation and differentiation is subject to variations influenced by cellular heterogeneity, genetic variants, and environmental factors [97]. The mechanisms governing cellular responses to mechanical stimulation remain incompletely understood across various cell types and tissues, necessitating further investigations for the standardisation of procedures and the definition of parameters to achieve ideal mechanical stimulation in diverse applications [98]. Despite current shortcomings, employing mechanical stimuli provides advantages in imitating physiological settings, increasing cell proliferation, directing cellular differentiation, and promoting tissue maturation [99].

## 8. Utilizing Photobiomodulation as a Biophysical Stimulus to Enhance Cellular Differentiation and Proliferation

The use of light, whether coherent or incoherent, in the visible and near-infrared (NIR) spectrum is termed PBM, which activates endogenous chromophores, leading to both photochemical and photophysical reactions [100]. Although the exact mechanisms are not fully understood, PBM stimulates cell signalling cascades and effector molecules, resulting in alterations in cell performance [101]. The “Cytochrome c Oxidase (CCO) Theory” proposes a biochemical reaction of PBM involving wavelengths between 600 and 1100 nm, wherein red or NIR light penetrates the cell membrane, targeting mitochondria and facilitating light absorption by cytochrome c within mitochondria [102]. This enzymatic chromophore aids in the electron transport chain during ATP production, ultimately inducing an increase in gene transcription within the cell nucleus, promoting DNA and RNA synthesis, and initiating cell proliferation [103]. However, the optimal method for enhancing proliferation and facilitating the differentiation of stem cells through PBM for clinical use is still under exploration [103,104,105,106]. Variations in cellular mechanisms due to photochemical procedures, dose dependency, limitations in cell lines for dosage, and the timing and frequency of exposure are currently under investigation to establish PBM parameters [101,107,108]. Studies suggest that PBM stimulates cell proliferation within specific wavelength and fluence ranges, such as between 660 and 850 nm and fluences of 5 to 10 J/cm^2^ [109]. Green light PBM within the range of 495 nm to 570 nm shows potential for improving cell differentiation, with low fluence contributing to enhanced involvement in cell differentiation [110]. However, high fluences may lead to increased levels of reactive oxygen species (ROS), cell damage, and cell death [111]. Treatment responses vary, with certain parameters stimulating cell proliferation and viability while others may inhibit it. Studies using low-power lasers with specific parameters have shown increases in angiogenic factors, decreases in apoptosis occurrence, regulation of cell adhesion and migration signals, and enhancements in cellular growth and proliferation without nuclear modifications [105,106,112,113,114,115].

## 9. Enhancing the Differentiation Potential of Adipose-Derived Stem Cells into Osseous Tissue through Three-Dimensional Cell Culture and Photobiomodulation

**Figure 4:**
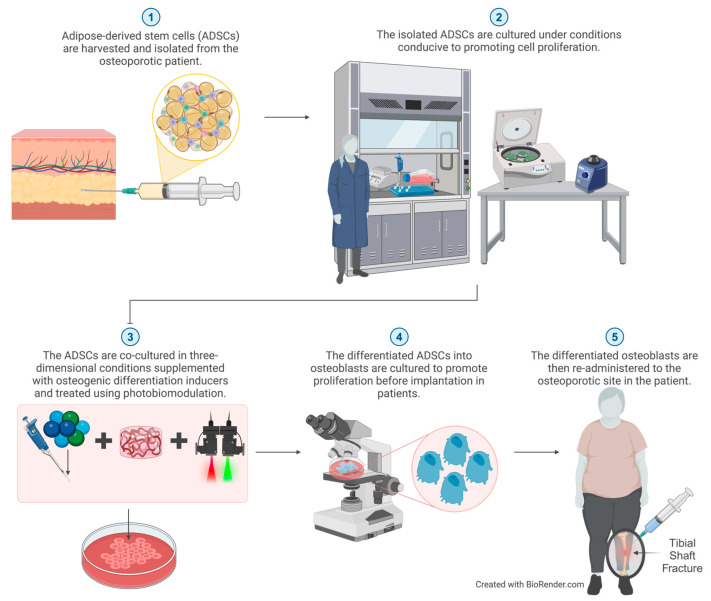
Theoretical clinical application utilizing three-dimensional cell culture settings and photobiomodulation treatment to induce adipose-derived stem cells to differentiate into osteoblasts, potentially offering regenerative therapy for osteoporosis.

The integration of PBM with 3D cell culture presents an innovative approach to facilitate the differentiation of ADSCs into osteoblasts and osteocytes for osseous tissue regeneration, as illustrated in Figure 4.

Three-dimensional cell culture methods, replicating the natural 3D architecture and cell-to-cell interactions of osseous tissue, play a pivotal role in promoting the differentiation of ADSCs into osteoblasts and osteocytes within a more physiologically relevant environment compared to conventional 2D cell culture [67]. By enhancing cell-to-cell and cell-to-matrix interactions, facilitating the interchange of signalling molecules, and promoting the differentiation and maturation of ADSCs into osteogenic cells, 3D cell culture critically contributes to the growth and functionality of osseous tissue [8]. Adipose-derived stem cells cultured in 3D conditions exhibit heightened osteogenic potential, evidenced by increased matrix mineralization deposition and elevated expression of osteogenic markers, ultimately leading to enhanced osseous tissue formation [116]. Incorporating PBM into the 3D cell culture model introduces an additional dimension to this approach [117]. Photobiomodulation triggers various cellular pathways, including the stimulation of the mitochondrial respiratory chain and alteration of the cytoskeleton, ultimately leading to increased proliferative activity and directed cell differentiation [118]. Optimal wavelengths for PBM fall within the optical window (450 to 1100 nm), enabling light to penetrate tissues and water-containing structures, making it suitable for tissue engineering and regenerative medicine applications [119,120]. Despite the potential of PBM, its application in 3D systems is intricate due to the diverse nature of 3D setups, irradiation sources, and protocols. Recent studies have explored the synergistic impact of PBM on ADSC proliferation and differentiation within a 3D cell culture model, demonstrating promising results [116,118,121]. Photobiomodulation, administered using specific parameters, significantly enhanced the proliferation rates of ADSCs without inducing cytotoxic effects, highlighting its potential as an augmentation strategy for 3D cell culture environments [121]. Additionally, in vivo studies have shown that the application of PBM, combined with ADSC-loaded hydrogels, promotes bone regeneration, and stimulates ADSCs for osteogenic differentiation, offering a potential solution for bone defect reconstruction [116,122]. Overall, the integration of PBM with 3D cell culture represents a novel and promising approach for advancing bone tissue engineering and regenerative medicine, with further research poised to unlock its full clinical potential.

## 10. Conclusions and Future Recommendations

In conclusion, exploring osteoporosis treatment through regenerative medicine and tissue engineering presents both opportunities and challenges. The global prevalence of osteoporosis highlights the urgent need for effective therapies, as current treatments have limitations. Regenerative medicine, particularly stem cell-based therapies, shows promise for enhancing bone regeneration but faces obstacles such as cell sourcing and immune rejection. Tissue engineering offers innovative solutions through multidisciplinary approaches, integrating stem cells, biomaterials, and PBM for tissue creation. Standardizing protocols and overcoming regulatory hurdles are crucial for clinical translation. Adipose-derived stem cells are promising but require strategies to manage their adipogenic tendencies. Three-dimensional cell culture techniques, including scaffolds and bioprinting, offer ways to replicate tissue complexity, but optimization is essential for physiologically relevant outcomes. Photobiomodulation can enhance cell function but needs standardised protocols. Addressing these gaps through collaborative efforts, rigorous studies, and adherence to ethical standards is vital for advancing regenerative medicine in osteoporosis treatment, promising to revolutionise management and improve the quality of life for millions worldwide.

## Figures and Tables

**Table 1:** An exploration of current therapeutic approaches employed in the treatment of osteoporosis.

Treatment Strategy	Example	Formulation	Mechanism	Side Effects	Reference
Bisphosphonates	Ibandronate, alendronate, zoledronate, risedronate, pamidronate, etidronate, and strontium ranelate	Oral/parenteral	Enhances osteoblast function while preventing bone resorption by attaching to surfaces where the breakdown of bone occurs.	Atypical fractures in the femur, gastrointestinal disturbances, osteonecrosis of the jaw, renal complications, thromboembolism, and an elevated likelihood of experiencing a myocardial infarction.	[29]
Hormone Replacement Therapy	17β-oestradiol, esterified and conjugated oestrogens	Oral/transdermal	Promotes osteoclast apoptosis.	Increased risks of breast cancer, stroke, and thromboembolism.	[30]
Selective Oestrogen Receptor Modulators	Raloxifene, arzoxifene, and calcitonin	Oral/parenteral intranasal oral	Replicates the impact of oestrogen on bone and suppresses bone resorption.	Occurrences of thromboembolic events, strokes in women who are postmenopausal and have cardiovascular disease. Furthermore, prolonged usage increases the risk of prostate cancer.	[31]
Parathyroid Hormone Analogues	Teriparatide	Parenteral	Stimulates the generation of osteoblasts and enhances the viability of existing osteoblasts.	While teriparatide has few side effects, its high cost and the necessity for subcutaneous injections are major disadvantages.	[32]
Receptor Activator of Nuclear Factor-Kappa B Ligand Inhibitors	Denosumab	Parenteral	Attaches to the receptor activator of nuclear factor-kappa B ligand, hindering the activity of osteoclasts.	Osteonecrosis, cystitis, and hypocalcaemia.	[33]

**Table 2:** The characteristics of cells utilised in tissue engineering.

Stem Cell Type	Source	Differential Potential	Differentiated Cells	Proliferation Potential	Allogenic/Autologous	Rejection Potential	Ethical Conflict	Clinical Drawback	Reference
Embryonic Stem Cell	Embryo	Pluripotent	Every cell type	Pinnacle	Allogenic	Probable	Numerous	Potential for tumour formation and supply volatility.	[51]
Induced Pluripotent Stem Cell	Transgenic somatic cells	Pluripotent	Every cell type	Pinnacle	Allogenic and autologous	Allogenic: Probable Autologous: Improbable	Minimal	Potential for tumour development, challenges in ensuring quality control.	[52]
Adult Stem Cell	Adult tissues	Multipotent	Restricted cell types	Limited	Allogenic and autologous	Allogenic: Probable Autologous: Improbable	By no means any	No identifiable risks.	[53]

**Table 3:** Techniques commonly used in three-dimensional cell culture.

Technique	Material	Advantages	Shortcomings	Reference
Scaffold-based System	Biological constituents, polymers, or ceramics.	Offers structural support and architectural framework for cell growth and organization, closely resembling the extracellular matrix.	Limited control over scaffold properties may hinder full replication of the native tissue microenvironment, necessitating intricate fabrication techniques.	[69]
Hydrogel-based Approach	Natural and/or synthetic polymers.	Provides adjustable mechanical properties, mimicking the native tissue extracellular matrix, and supporting cell adhesion, proliferation, and differentiation.	Lacks mechanical stability, with restricted control over degradation kinetics, potentially leading to compromised long-term viability.	[47]
Spheroids	Cell aggregates.	Enhances cell-to-cell interactions while replicating multicellular organization and the microenvironment.	Limited control over size, potential for central necrosis, and heterogeneous cellular distribution are notable concerns.	[88]
Organoids	Self-organising cell clusters.	Replicates both tissue architecture and function, serving as a platform for disease modelling and drug testing alike.	The complexity and variability inherent in organoid formation present significant challenges to reproducibility, alongside limitations in scalability.	[89]
Bioprinting	Natural and/or synthetic biomaterial bio inks and cell aggregates.	Enables precise spatial control over cell deposition, thereby facilitating the fabrication of intricate three-dimensional structures with ease.	The constrained selection of printable biomaterials, coupled with difficulties in achieving vascularization and seamless integration of printed constructs, contributes to the high cost and technical intricacy of the process.	[90]

## Data Availability

The literature review relied on databases such as PubMed, Scopus, Web of Science, and Google Scholar, with findings supported by previously reported studies and datasets cited in the manuscript and available within its references. No new data were generated or analysed in this study.
